# Solvent-free formation of a disulfide/sulfone polymer network for salt-driven atmospheric water harvesting

**DOI:** 10.1039/d6qm00173d

**Published:** 2026-05-28

**Authors:** Joseph J. Dale, Mathilde Gerbaud, Robert T. Woodward

**Affiliations:** a Institute of Materials Chemistry and Research, Faculty of Chemistry, University of Vienna Währinger Straße 42 1090 Vienna Austria Joseph.dale@univie.ac.at robert.woodward@univie.ac.at

## Abstract

The ongoing water crisis requires the development of functional materials that can tap into the atmospheric water reservoir. Sorption-based atmospheric water harvesting using porous materials presents a promising solution. Many porous, crystalline networks have been investigated thus far, however, simple, hydrophilic polymers may prove a promising branch of sorbents for versatile water capture. Here, poly-PETMP is produced *via* thiol self-condensation in a bulk synthesis at room temperature in the absence of organic solvents. The resulting poly-PETMP is then loaded with calcium chloride to yield a sorbent with an impressive water sorption capacity of 1.34 g g^−1^ at 90% relative humidity (RH) and 0.29 g g^−1^ at 30% RH, while retaining a consistent performance over repeated sorption/desorption cycles.

## Abstract 2

The ongoing water crisis requires the development of functional materials that can tap into the atmospheric water reservoir. Sorption-based atmospheric water harvesting using porous materials presents a promising solution.

## Graphical abstract

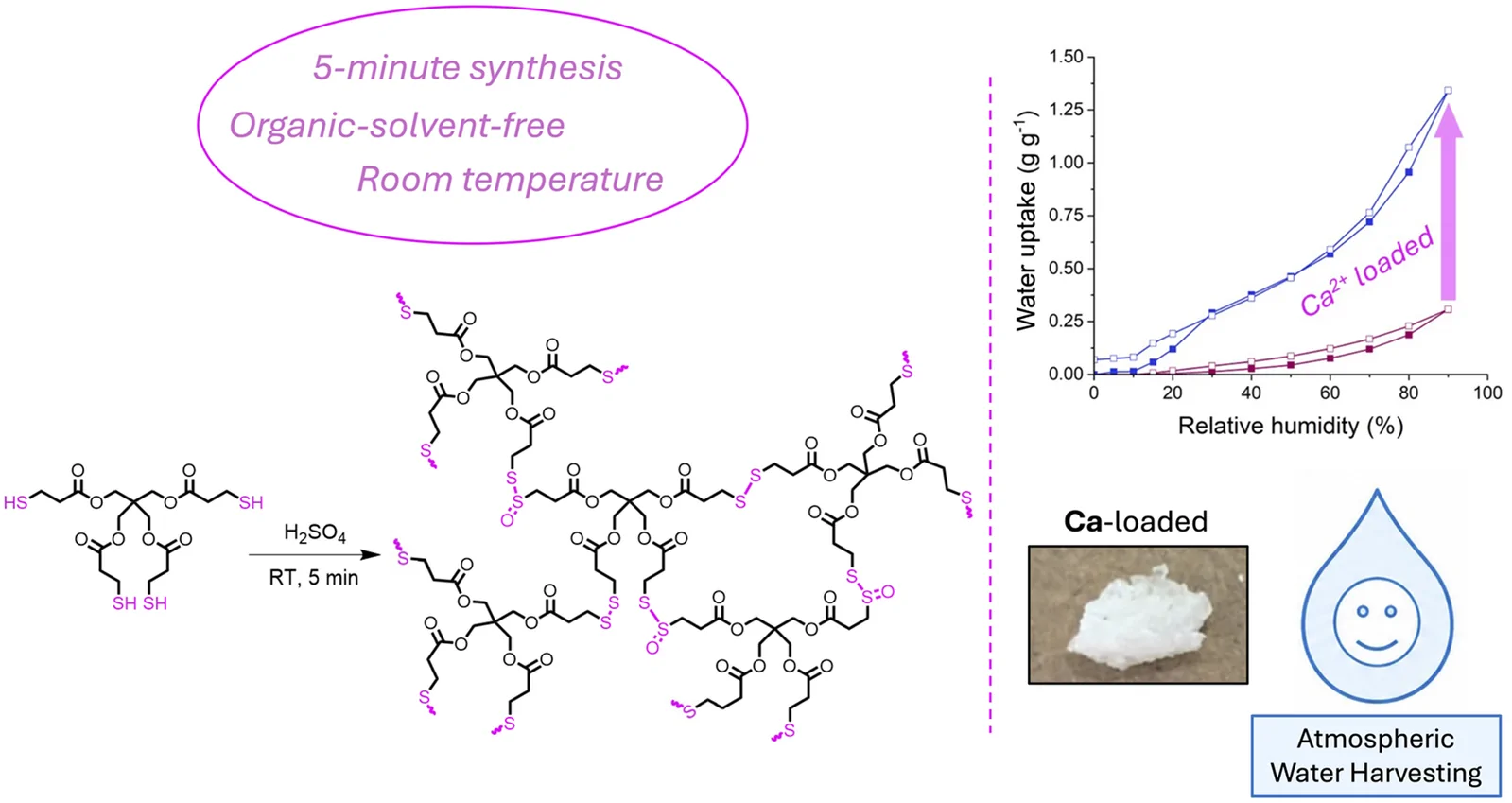

## Introduction

Climatological natural disasters, fueled in part by climate change and population growth, have been escalating around the world, causing devastation to living spaces, agriculture, and human lives.^1^ The World Health Organisation (WHO) predicts that “…as many as 700 million people are at-risk of being displaced as a result of drought by 2030”.^2^ The water crisis, in which clean, safe, and affordable freshwater supplies are insufficient to meet demands, requires innovative, low-cost solutions.^3^ Tapping into the water content of the atmosphere, which holds up to six-times the volume of water in all the rivers of the world combined,^4^ has the potential to tackle the water crisis.

Atmospheric water harvesting (AWH) sorbents are generally hydrophilic networks that can extract water from air.^5,6^ Examples of material classes under investigation for AWH are MOFs,^7^ COFs,^8^ hydrogels,^9^ and polymer networks.^10^ These structures are typically porous and contain hydrophilic chemical moieties within the structure to incite water sorption. Hygroscopic salts are often incorporated into AWH materials to enhance their hydrophilicity, drastically increasing their water uptake capacities and shifting adsorption to the low relative humidity regime (10–30% RH).^11^ Water sorption in this regime is crucial for AWH sorbents, as water scarcity is most prevelant in dry and arid regions.^12^ Indeed, Tian *et al.* reported a 30 wt% LiCl-loaded MOF-303 that exhibited a water sorption of 0.61 g g^−1^ at 30% RH and 25 °C.^13^ Xu *et al.* detailed their LiCl@MIL-101(Cr) material that can adsorb 0.77 g g^−1^ under similar conditions.^14^ An *et al.* explored a CaCl_2_ loaded MOF-808 with a water harvesting potential of 0.56 g g^−1^ at 30% RH and 25 °C, compared to 0.08 g g^−1^ in its non-salt loaded equivalent.^15^ While materials such as those listed are promising, exhibiting exceptional adsorbing properties at low RH in some cases, they are not without issue. Materials such as MOF-303^16^ and MOF-801^17^ have previously required lengthy solvent washing protocols (a green synthesis of MOF-303 has since been reported by Zheng *et al.*,^18^ and of MOF-801 by Hashjin *et al.*^19^), while networks such as AB-COF^20^ and hypercrosslinked polymer SHCP-10^21^ require the use of environmentally damaging solvents such as 1,2-dichlorobenzene or 1,2-dichloroethane. Exposure to chlorinated solvents can lead to respiratory problems, including asthma and bronchitis,^22^ hence research is underway to find safe and sustainable alternatives in the synthesis of hypercrosslinked polymers.^23^ While methods to process and recycle solvents are available,^24^ these processes can be energy-intensive, rendering the complete elimination of organic solvents in material synthesis more favourable.

**Fig. 1:**
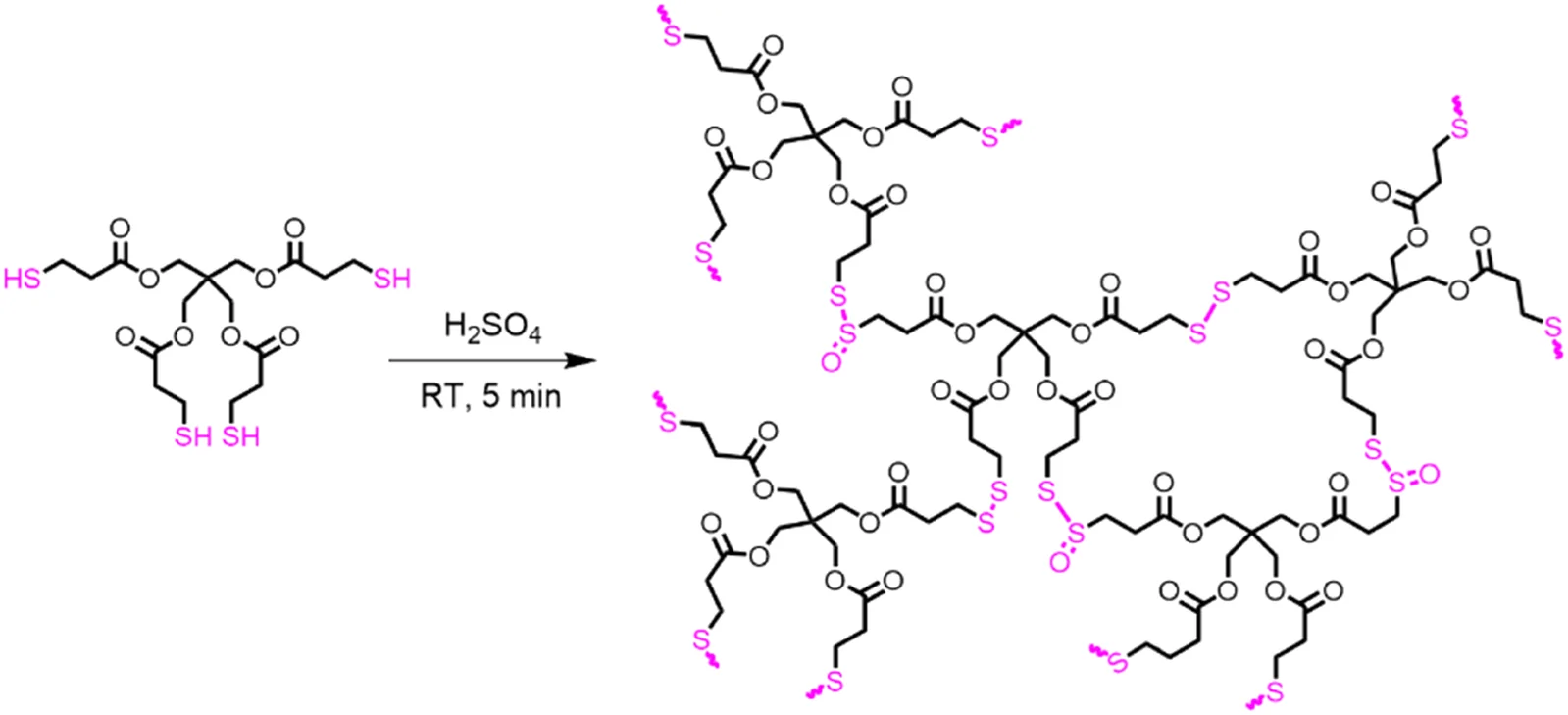
Synthetic route to, and suggested structure of, poly-PETMP.

Sorbents for AWH should offer fast adsorption/desorption kinetics, high overall capacities, and chemical and thermal robustness;^25^ however, ease of synthesis and green credentials must also be at the forefront of material design. Here, we utilise the acid-catalysed thiol self-condensation of pentaerythritol tetrakis(3-mercaptopropionate) (PETMP) *via* a rapid, solvent-free synthesis to produce a crosslinked polymer matrix (poly-PETMP) (Fig. 1). Poly-PETMP contains disulfone bridging bonds and ester linkages, which act as dual hydrophilic points and coordinating sites for hydrophilic metal centers. A hygroscopic salt, CaCl_2_, is then incorporated into the polymer structure, with Ca^2+^ ions acting as nucleating points for water cluster formation. The salt-loaded poly-PETMP demonstrates a water sorption capacity of 1.34 g g^−1^ at 90% RH and 25 °C, while also exhibiting an impressive water sorption of 0.29 g g^−1^ at 30% RH and 25 °C. Poly-PETMP represents a new brand of flexible, non-porous polymeric material for AWH, with impressive water sorption capacity, consistent performance over repeated adsorption/desorption cycling, and a green synthesis requiring no organic solvents.

## Results and discussion

To produce poly-PETMP, sulfuric acid was added to neat PETMP over stirring. The viscosity of the solution increased rapidly, forming a yellow gel within seconds that prevented agitation. The addition of NaOH solution caused the gel to expand into a white foam-like structure (Fig. S1), yielding poly-PETMP, which was washed with additional NaOH solution to deprotonate thiols and prevent foul odours. The polymer was dried in an oven at 80 °C for 24 h to yield a white solid, poly-PETMP (suggested structure in Fig. 1). Poly-PETMP was a brittle, hard material when oven dried, and malleable with a rubber-like quality when hydrated. Calcium-loaded poly-PETMP (poly-PETMP-Ca) was prepared by adding 100 mg poly-PETMP to 50 mL of a saturated CaCl_2_ solution and stirring for 6 h at room temperature before filtering and drying in an oven at 80 °C for 24 h.

**Fig. 2:**
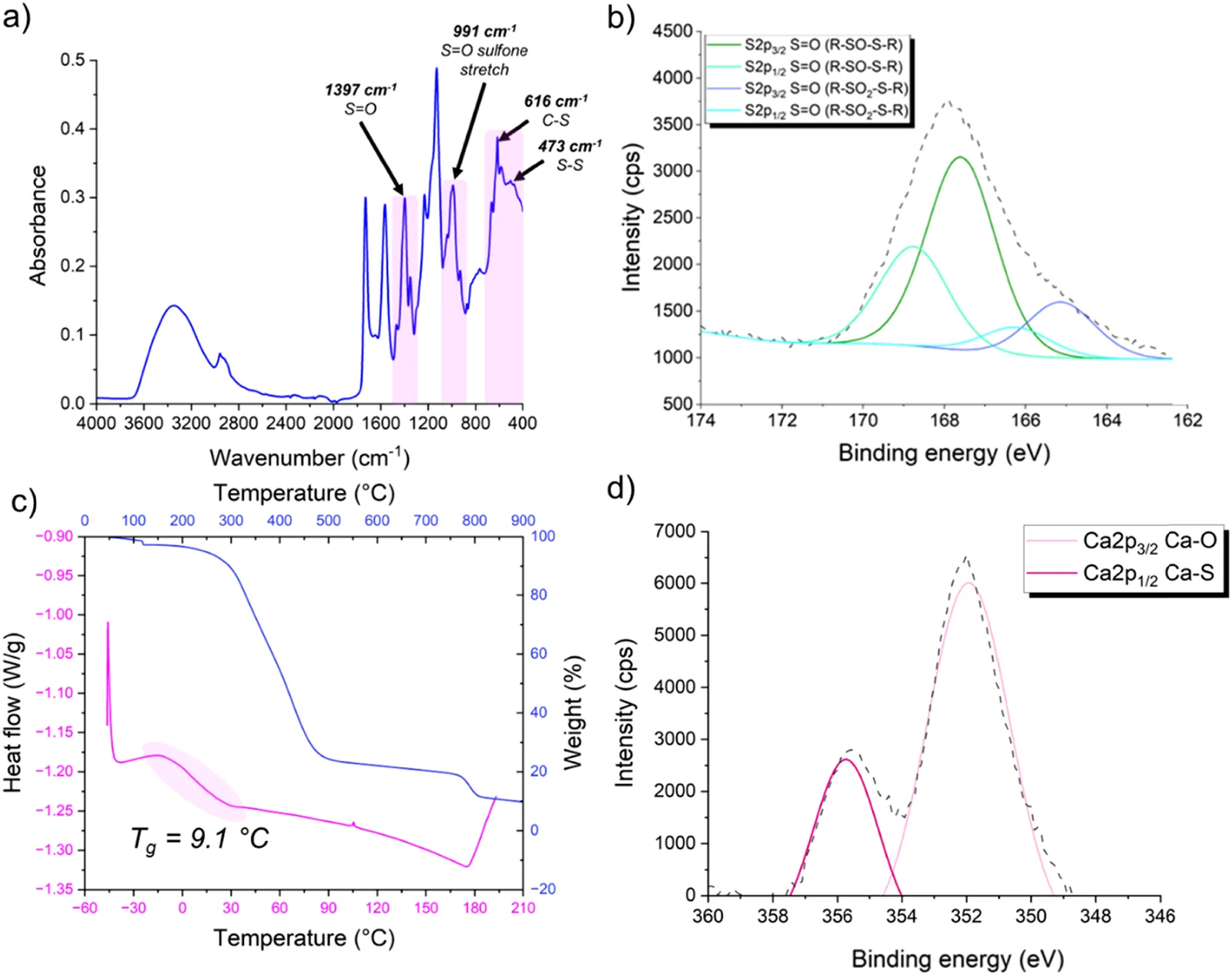
(a) FTIR spectrum of poly-PETMP highlighting S–S bond stretching, and sulfonic acid S=O stretching. (b) XPS S 2p spectrum of poly-PETMP. (c) TGA (blue) and DSC (pink) thermograms of poly-PETMP, showing the decomposition transitions and glass transition temperature, *T*_g_, for poly-PETMP. (d) XPS Ca 2p spectrum of poly-PETMP-Ca.

Fourier transform infrared (FTIR) analysis (Fig. 2a) showed no thiol stretching band, indicating the consumption of PETMP's thiol groups or their deprotonation by NaOH washing to form R–SNa. The band at 473 cm^−1^ is assigned to S–S bond stretching, suggesting that the polymerisation mechanism is an acid-catalysed thiol self-condensation in which sulfuric acid driven oxidation of thiols leads to sulfenic acids, before self-condensation and release of water to form disulfide bridges. Further oxidation of disulfides to disulfones/sulfoxides then occurs, as evidenced by the S=O sulfone (R–SO–S–R) stretch at 991 cm^−1^. A peak at 616 cm^−1^ shows the retention of the C–S bond, while peaks at 1132 cm^−1^ and 1726 cm^−1^ correspond to the C–O and C=O bonds, respectively, confirming ester group retention. A broad O–H band at 3000–3600 cm^−1^ is assigned to the asymmetric stretch of water molecules, indicating the presence of water in the polymer matrix.

X-ray photoelectron spectroscopy (XPS) corroborated the FTIR analysis, with high resolution S 2p spectra showing S 2p_1/2_ and S 2p_3/2_ bands at 168.8 eV and 167.6 eV, respectively, assigned to the R–SO_2_–S–R sulfone bond, while the bands at 166.3 eV and 165.1 eV correspond to the R–SO–S–R bond, confirming oxidation of disulfide linkages (Fig. 2b).^26^ The lack of S=O sulfonic acid bonding in the XPS data suggests that oxidation beyond sulfone groups does not occur. A small shoulder at ∼163.0 eV is suggested to be the S–S disulfide bond, however, confident deconvolution was not possible in this region. CHNS–O elemental analysis of poly-PETMP was in rough agreement with the theoretical mass of each element (Table S1), with an increased oxygen content due to water sorption and a residual mass of 2.5% suggested to be Na^+^ ions after washing.

Differential scanning calorimetry (DSC, Fig. 2c and Fig. S2) confirmed polymer formation with a glass transition temperature (*T*_g_) appearing at 9.1 ± 0.6 °C. The polymer proved insoluble in a variety of solvents across a range of polarities (Table S2), indicative of a crosslinked structure. Thermogravimetric analysis (TGA) at 120 °C for 30 min under a N_2_ atmosphere (Fig. 2c) yielded a mass loss of 1.3 ± 0.5 wt%, attributed to water loss. After this isothermal drying step, poly-PETMP showed two decomposition steps. The first, arising at 310 ± 7 °C, yielded a mass loss of 73.9 ± 0.4%, designated as the overall breakdown of the polymeric structure including disulfones and esters. The second decomposition, observed at 766 ± 11 °C, yielded a mass loss of 8.6 ± 0.1% and is likely the breakdown of residual carbonaceous material. The polymer displayed no significant porosity, exhibiting a BET apparent surface area of <1 m^2^ g^−1^ (N_2_ sorption isotherm provided in Fig. S3). Scanning electron microscopy (SEM) images confirmed a non-porous morphology (Fig. S4). XPS confirmed successful calcium loading into the poly-PETMP-Ca structure with peaks at 354.1 eV and 375.5 eV in the Ca 2p spectrum (Fig. 2d), ascribed to Ca–O and Ca–S bonds, respectively, as calcium coordinated to lone pairs on O/S. After calcium loading, CHNS–O analysis yielded a total mass of 47.08 wt%, while X-ray fluorescence spectroscopy confirmed a calcium content of 11.9 wt%. The remaining mass is attributed to the formation of CaSO_4_ upon combustion, as well as residual chloride and sodium. We conducted a swelling study (Table S3) on poly-PETMP in water, ethanol, and hexane, representing solvents of different polarities. Swelling decreased with decreasing polarity, with hexane unable to penetrate the poly-PETMP structure, thus demonstrating the hydrophilicity of the network.

**Fig. 3:**
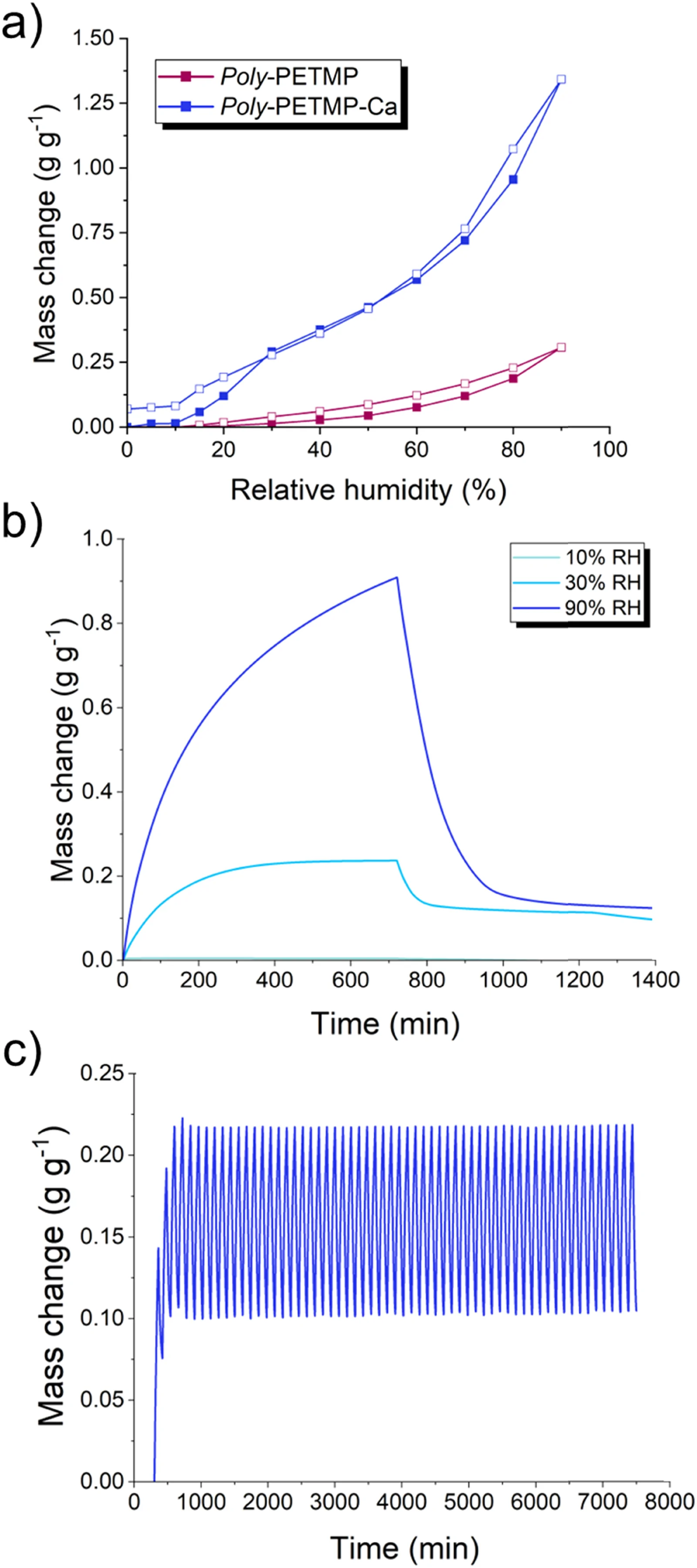
(a) Water sorption isotherms of poly-PETMP (blue) and poly-PETMP-Ca (pink) at 25 °C. (b) Time-dependent water adsorption and desorption of poly-PETMP-Ca at 10, 30, and 90% RH and 25 °C. (c) Cycling of poly-PETMP-Ca over 60 water sorption–desorption cycles at 25 °C. Uptake and desorption steps were each held at 40% and 0% RH, respectively, for 1 h.

A water sorption isotherm of poly-PETMP was measured at 25 °C using dynamic vapour sorption (DVS) (Fig. 3a) and exhibited a modest total water sorption capacity of 0.31 g g^−1^ at 90% RH. After loading with CaCl_2_, the water sorption capacity of poly-PETMP-Ca increased dramatically to 1.34 g g^−1^ under the same conditions. Photographs of poly-PETMP-Ca before and after absorption at 90% RH for 24 h are provided in Fig. S5. Mild hysteresis in the isotherm, and incomplete desorption at 0% RH, are due to the strong interactions of water molecules in the hydration shells of Ca^2+^ ions. At 30% RH, poly-PETMP-Ca retained an impressive water uptake of 0.29 g g^−1^, demonstrating effective water sorption even under the dry conditions of interest in AWH applications. A two-step adsorption profile is observed, with a sharp uptake from 10–30% RH, before seeming to change to a different sorption mechanism beyond 30% RH. We tentatively suggest a mechanistic explanation of the water sorption behaviour; at low RH, sorption is driven by Ca^2+^ hydration shell filling, while adsorption above 30% RH is driven by cluster formation and growth around the hydrated Ca^2+^ ions. Calcium-coordinated sites may provide highly hydrophilic points that allow nucleation of water clusters in N–S(iii)-type behaviour^27^ (non-S-shaped isotherm featuring slow uptake at low RH, increasing in uptake rate at high RH). As RH increases, N–S(iii) uptake continues in a new regime, with cluster growth occurring, driven by water–water interactions.^28^ Finally, at high RH, surface condensation yields a steep uptake in water sorption. We provide a comparison table to other reported amorphous polymers and calcium containing materials in Table S4. While not directly competitive with other Ca^2+^ containing materials, poly-PETMP-Ca presents a high absorption capacity for a simple polymer structure prepared in a rapid bulk synthesis.

The rates of water adsorption and desorption were determined at 10, 30, and 90% RH by holding poly-PETMP-Ca under each condition for 12 h before returning to 0% RH for a further 12 h for desorption (Fig. 3b). At 10% RH, little to no adsorption was observed, aligning with the water sorption isotherm. At 90% RH, a maximum capacity of 0.91 g g^−1^ was observed, indicative of the adsorption not reaching equilibrium and suggesting that surface condensation is slow in poly-PETMP-Ca due to the lack of measurable surface area. Incomplete desorption was observed in all cases, aligning with the isotherm data, and ascribed to the strong attractions of water molecules in the Ca^2+^ hydration shells preventing complete dehydration at 0% RH. The water diffusivity at 30% RH and 25 °C, derived from Fick's law of diffusion, was determined to be 6.44 × 10^−11^ m^2^ s^−1^, thus explaining the slow adsorption rate of poly-PETMP-Ca, and why the sample has not reached the maximum adsorption capacity after 12 h of exposure to 90% RH.^29^ The estimated diffusivity is notably lower than that of other CaCl_2_ containing materials,^30,31^ a contributing factor again being a lack of porosity. We note the inaccuracy in this calculation, however, as it assumes that the sample is an ideal film, rather than a bulk polymer monolith.

The stability of poly-PETMP-Ca with respect to repeated adsorption/desorption was assessed using DVS over 60 cycles *via* a humidity swing between 0 and 40% RH with a 1 h isothermal hold between each step (Fig. 3c). Poly-PETMP-Ca demonstrated exceptional stability over 60 cycles, with consistent adsorption and desorption after conditioning. The uptake during cycling is noted to be lower than that measured in the water sorption isotherm, ascribed to the slow adsorption rate. A dry sample of poly-PETMP-Ca was held at 90% RH for 8 h before it was dried again and held once more for 8 h at 90% RH. The absorption capacity remained unchanged (1st cycle = 0.67 g g^−1^, 2nd cycle = 0.68 g g^−1^), demonstrating the retention of poly-PETMP-Ca's water sorption properties after exposure to high RH (Fig. S9). Cycling data demonstrates that poly-PETMP-Ca is a robust and consistent water harvesting material. However, we note that mechanical stability may be altered under adsorption/desorption conditions and seek to investigate this in our future work.

**Fig. 4:**
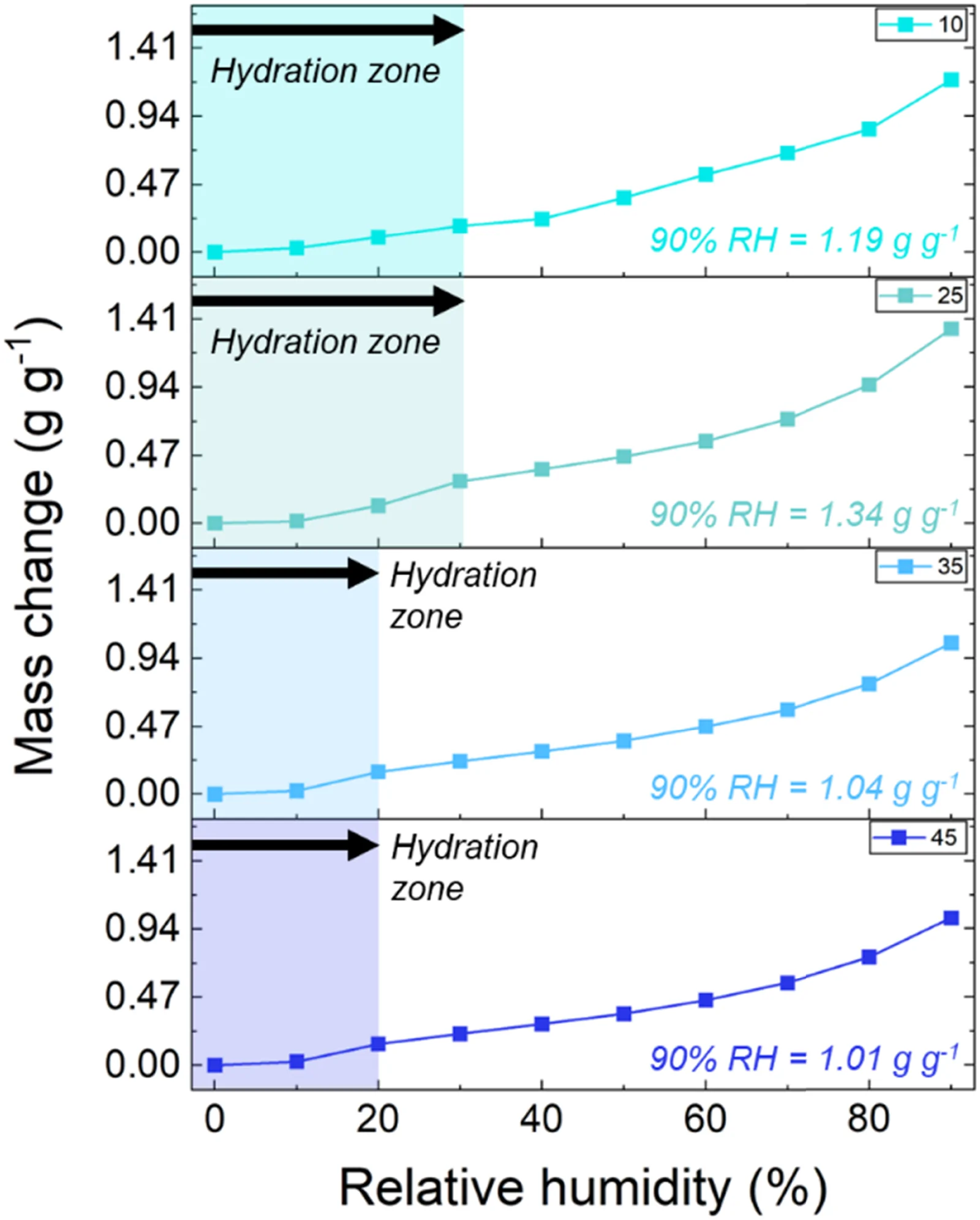
Water sorption isotherms of poly-PETMP-Ca measured at 10, 25, 35, and 45 °C, demonstrating the shift in the Ca^2+^ hydration shell-filling region.

The isosteric heat of adsorption (Δ*H*_Ads_) of poly-PETMP-Ca was calculated to be *Q*_st_ = 47.5 kJ mol^−1^ from the isotherms at 25, 35, and 45 °C (Fig. S6) using the Clausius–Claperyron equation. The Δ*H*_Ads_ of bulk water is *Q*_st_ = 44 kJ mol^−1^.^32^ The higher Δ*H*_Ads_ of poly-PETMP-Ca explains the lack of complete desorption, as the interactions of water molecules with the Ca^2+^ first hydration shell are stronger than in bulk water. The hydrated Ca^2+^ ion exists in an eight-coordinate antiprismatic structure, with an average Ca–O bond length of 2.476 Å and the presence of a second hydration shell.^33,34^ For comparison, the O–O intermolecular bond length of bulk water is reported to be 2.902 Å.^35^ Thus, the shorter bond length requires a greater energy input to break and release the water molecules. To investigate the effects of temperature on water capacity, water sorption isotherms conducted at temperatures ranging between 10–45 °C were compared (Fig. 4). We theorise that a polymer *T*_g_ below room temperature may convey a higher degree of chain mobility, allowing for greater water cluster growth. A water sorption isotherm completed at 10 °C (the lowest temperature available for the instrument) yielded a mass uptake at 90% RH of 1.19 g g^−1^ compared to 1.34 g g^−1^ at 25 °C (Fig. S7 and S8). We posit that, at 10 °C, polymer chain mobility decreases and thus the maximum possible water cluster growth reduces with a reduced swellable space. Significant hysteresis is observed at 10 °C, with 0.18 g g^−1^ of water remaining within the polymer sample.

The absolute capacity measured at 90% RH decreased with increasing temperature, with 1.04 g g^−1^ and 1.01 g g^−1^ measured at 35 °C and 45 °C respectively. At 30% RH, a similar trend is followed, with 0.18, 0.29, 0.23, and 0.22 g g^−1^ recorded for 10, 25, 35, and 45 °C, respectively. The two-step adsorption profile, observed to change sorption mechanisms at 40% RH at <25 °C, is shifted to lower RH (30%) at elevated temperatures. A faster rate of Ca^2+^ ion hydration could occur at higher temperatures,^36^ while the decrease in sorption capacity at high RH with increasing temperature (Fig. S7 and S8) is suggested to be due to the decrease in calcium hydration number with increasing temperature, as detailed by Zavitsas.^37^ A decrease in hydration number would decrease the number of water molecules occupying the primary hydration shell, reducing the sorption capacity and initial water sorption driving force. At lower temperatures, the capacity may decrease due to the decrease in polymer chain mobility. At high temperatures, the shift in hydration region would therefore align with the decrease in hydration number, and an earlier onset of water cluster formation/growth. Therefore, 25 °C is suggested as the optimum sorption temperature due to the greater potential of Ca^2+^ ion hydration *vs.* polymer chain mobility.

To assess the potential leaching of calcium from poly-PETMP-Ca upon uptake-desorption, we conditioned a sample at 75% RH for 24 h before drying in an oven at 80 °C. TGA of the pristine and dried sample showed no change in the residual mass of Ca-based species, suggesting no significant leaching occurred (Fig. S10). We propose that poly-PETMP, while demonstrated here for AWH, is a simple system that merits further investigation. A bulk, room temperature polymer synthesis is attractive for the design of functional materials; however, we note some of our scale-up considerations:

1. The requirement of concentrated sulfuric acid, when handled in large quantities, is hazardous.

2. Thiols are known for their potent odour. Large quantities of thiols should therefore be handled in a high-velocity extracted area.

3. Sulfur chemistry can often produce dangerous byproducts *e.g.* H_2_S gas. However, the theoretical sulfur content for poly-PETMP is in good agreement with the measured sulfur content, suggesting no significant evolution of dangerous S-containing species.

4. PETMP monomer is a viscous liquid, thus agitation at scale (conferred by impeller or other system) must be considered and optimised.

## Conclusions

A crosslinked poly-PETMP network has been prepared by a facile, bulk acid-catalysed self-condensation of the tetra-thiol monomer PETMP. The synthesis occurs within minutes, requires no additional solvent, and uses only sulfuric acid as polymerisation catalyst, addressing Green Chemistry Principles 2, 5, 6, and 7.^38^ Poly-PETMP was loaded with Ca^2+^*via* binding at a variety of potential coordination sites to increase the overall network hydrophilicity, yielding an impressive total water sorption capacity of 1.34 g g^−1^ in a non-porous, amorphous polymer material. The hydration of Ca^2+^ ions, combined with the flexible nature of a polymer structure with a *T*_g_ below room temperature, and hydrophilic disulfone crosslinks^39^ allow for significant adsorption of water even at low RH. Poly-PETMP-Ca demonstrated robust water sorption properties, cycling consistently over 60 adsorption/desorption cycles. It is suggested that a balance between polymer chain mobility and Ca^2+^ hydration number is required for optimal water sorption capacity. It is hoped that this research represents the start of a new generation of hydrophilic, rapidly produced polymer materials for AWH with minimal waste generation. We note that poly-PETMP-Ca, while demonstrating promising sorption properties, is not yet competitive with the state-of-the-art Ca^2+^-containing materials (Table S4). However, poly-PETMP-Ca shows promise in the drive to the commercial implementation of AWH sorbents owing to its simple, organic-solvent-free preparation and processability. While sophisticated porous networks may currently be the most promising materials for AWH, polymeric networks with rapid and simple syntheses may prove invaluable for fast practical application and for finding a solution to the water crisis.

## Conflicts of interest

There are no conflicts to declare.

## Supplementary Material

QM-010-D6QM00173D-s001

## Data Availability

The data supporting this article have been included as part of the supplementary information (SI). Supplementary information: experimental procedures, additional characterisation data (FTIR, XPS, CHNS analysis, DSC, TGA, N_2_ sorption), dynamic vapour sorption measurements, solubility studies, and supporting tables and figures (Tables S1–S4 and Fig. S1–S10). See DOI: https://doi.org/10.1039/d6qm00173d.
