# Supplementary material for: Solvent-free formation of a disulfide/sulfone polymer network for salt-driven atmospheric water harvesting
Source: Mater Chem Front. 2026 May 28. Online ahead of print. doi: 10.1039/d6qm00173d (PMC13231942; doi:10.1039/d6qm00173d)
Supplement: QM-OLF-D6QM00173D-s001 [file QM-OLF-D6QM00173D-s001.pdf]

Supplementary Information (SI) for Materials Chemistry Frontiers.
This journal is © the Partner Organisations 2026

Solvent-Free Formation of a Disulfide/Sulfone Polymer Network For Salt-Driven Atmospheric Water Harvesting – Supplementary Information

Joseph J. Dale,[1,*] Mathilde Gerbaud,[1] Robert T Woodward[1,*]

[1] Institute of Materials Chemistry and Research, Faculty of Chemistry, University of Vienna, Währinger Straße 42, 1090, Vienna, Austria

* Email: Joseph.dale@univie.ac.at, robert.woodward@univie.ac.at

Materials

Pentaerythritol tetrakis(3-mercaptopropionate) (>95%), calcium chloride dihydrate (>99 %) and sodium hydroxide pellets (>98 %) were purchased from Sigma-Aldrich. Sulfuric acid (>95%) was purchased from Fischer Scientific. All chemicals were used as received with no further processing.

Methods

To a 25 mL round bottom flask PETMP (1 mL, 2.62 mmol) was added and agitated by magnetic stirring at 400 rpm at room temperature. 0.2 mL sulfuric acid (95%) was added. The viscosity of the solution increased rapidly, within seconds forming a yellow gel that prevented agitation. NaOH solution (1 M, 20 mL) was added, and the gel expanded into a white foam. After 5 minutes the foam was filtered and washed with a further 100 mL NaOH solution. The sample was dried in an oven at 80 °C for 24 hours. All samples were prepared in triplicate.

Swelling tests were conducted by placing a known mass of *poly*-PETMP in 1 mL of solvent in a glass sample vial for 1 h. After removal, residual solvent was removed by placing the sample carefully on a paper towel. The polymer samples were then reweighed, and the swelling percentage was calculated according to equation 1. n = 3

$$SP = \left(\frac{Mass_{(Wet)} - Mass_{(Dry)}}{Mass_{(Dry)}}\right) x\ 100$$

Characterisation

Fourier transform infrared (FTIR) spectroscopy

FTIR was conducted using a Bruker Tensor II Spectrometer for 64 scans between 4000-400 cm^{-1} at a resolution of 4 cm^{-1}.

X-ray photoelectron spectroscopy (XPS)

XPS was performed using a Nexsa Photoelectron Spectrometer (Thermo Scientific). All measurements were performed using Al-Kα X-rays with a spot size of 400 µm. High-resolution spectra of calcium (Ca 2p 340–360 eV), sulfur (S 2p 157– 175 eV) were recorded with a resolution of 0.1 eV and a pass energy of 50 eV. Gaussian smoothing (FWHM (eV) = 2.0, Passes = 1) of the S 2p spectrum was conducted in Avantage S4 software (v5.9931, Thermo Fisher Scientific). In OriginPro 2024, baseline correction was conducted before Multiple Peak Fit deconvolutions using a Gaussian fit.

Elemental analysis (CHNS)

Elemental analysis was performed using a Eurovector EA 3000 CHNS-O Elemental Analyser. 1-2 mg of sample was weighed into tin vials (4×6 mm) and measured in triplicate. 1000 °C combustion temperature and 750 °C reduction temperature were applied in helium (99.999+) carrier gas.

Differential scanning calorimetry (DSC)

DSC was conducted by applying a heat-cool-heat cycle on a TA Instruments Discovery series instrument. Samples were prepared in Tzero aluminium pans. The samples were equilibrated at 20 °C before applying a heating cycle at 10 °C min^{-1} to 200 °C. A cooling cycle of 10 °C min^{-1} to -50 °C was applied, before a final heating cycle of 10 °C min^{-1} to 200 °C. A 1 minute isothermal step was included between each heating/cooling cycle.

Solubility studies

10 mg of *poly*-PTMP was placed in a glass sample vial containing 10 mL of solvent (solvents listed in Table S1 below). No dissolution was observed to have occurred after 24 hours.

Thermogravimetric analysis (TGA)

TGA was conducted on a TA Instruments Discovery TGA. Samples were heated at 10 °C min^{-1} to 120 °C before a 30 min isothermal drying step. After this a 10 °C min^{-1} heating rate was applied to a maximum of 900 °C under N_2 gas at a flow rate of 25 mL min^{-1}.

N_2 gas sorption

N_2 gas sorption was carried out on a TriStar II (Micromeritics Instrument Corporation) instrument at 77 K. Samples were degassed for 24 hours at 100 °C under N_2 gas flow prior to analysis using a FlowPrep 060 (Micromeritics Instrument Corporation). BET surface areas were calculated using the Brunauer-Emmett-Teller (BET) method on the adsorption branch between 0.05 - 0.2 P/P0.

Scanning electron microscopy (SEM)

Samples were dried for 24 h in an oven at 80 °C, prior to analysis. SEM images were captured using a Jeol JCM-6000 instrument operated at an acceleration voltage of 15 kV. Samples were loaded onto a carbon backed SEM stub before sputter gold coating was conducted using a Jeol JFC-1200 Fine Coater. Samples were coated for 20 s while maintaining an atmospheric pressure of 8 Pa. n = 1 for all samples.

Dynamic vapour sorption (DVS)

DVS was carried out on a DVS Discovery (Surface Measurement Systems). 10-20 mg of polymer was weighed into an aluminium sample pan and exposed to a variable RH atmosphere of DI water. Isotherm measurements were taken at 10% RH increments from 0-90% RH. Rate measurements were carried out by holding the polymer in RH environments of 30, 50, and 90% for 12 hours, with a 12 hour drying step between each adsorption. Cycling stability measurements were taken over 60 cycles alternating between 0 and 40% RH for 1 hour each. A 12 hour drying step at 0% RH was included before all analyses. All analyses were conducted at 25 °C.

Statistical analysis

All graph plotting was conducted in OriginPro 2024. Error bars represent standard deviation calculated in Microsoft Excel using at least three measurement repeats on discrete samples.

FTIR analysis was conducted using the OPUS 7.5 software. DSC and TGA were conducted using the TA Instruments Trios software. DVS was conducted using DVS control software. N_2 gas sorption analysis was carried out using a TriStar II (Micromeritics Instrument Corporation) software.

XPS data processing was conducted using the Avantage S4 software (v5.9931, Thermo Fisher Scientific) and OriginPro 2024. Gaussian smoothing (FWHM (eV) = 2.0, Passes = 1) of the high-resolution sulfur spectrum was conducted in Avantage S4 software (v5.9931, Thermo Fisher Scientific). In OriginPro 2024, baseline correction was conducted before Multiple Peak Fit deconvolutions using a Gaussian fit.

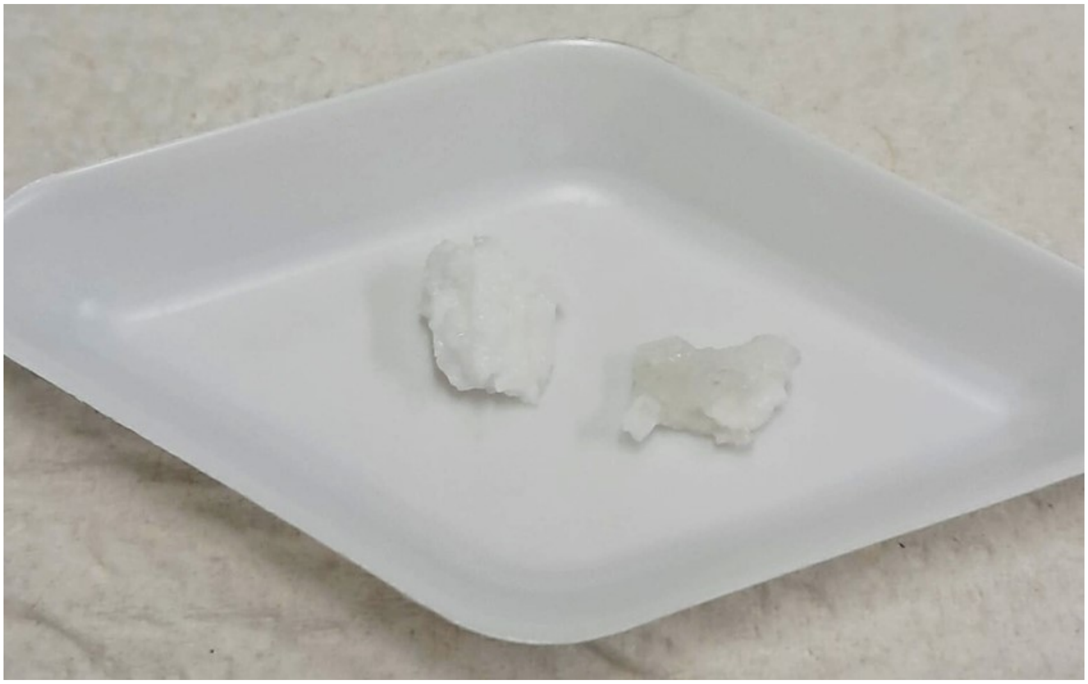

Figure S1. Picture of *poly*-PETMP.

Table S1. CHNSO elemental analysis of *poly*-PETMP, and *poly*-PETMP-Ca.

	% Mass		
**Element**	**Theoretical**	***poly*-PETMP**	***poly*-PETMP-Ca**
C	41.80	37.74	18.59
H	5.74	4.82	3.09
N	-	-	-
S	26.23	25.43	10.56
O	26.23	29.47	14.79
**Total**	**100**	**97.51**	**47.08**

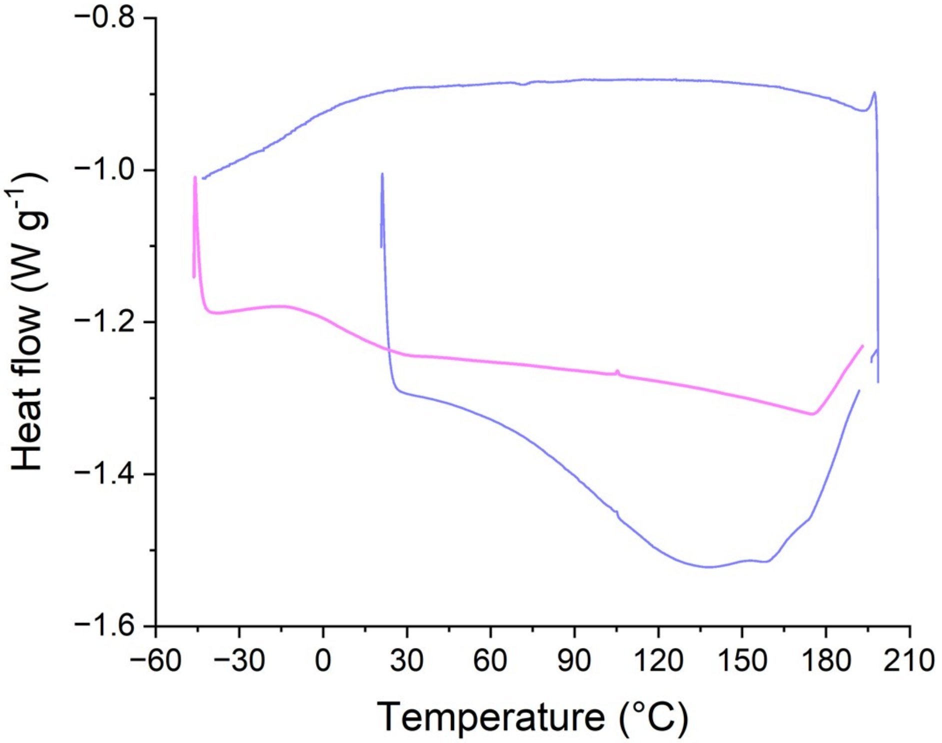

Figure S2. Complete heat-cool-heat cycle of *poly*-PETMP. Second heating curve shown here in pink.

Table S2. The solubility of *poly*-PETMP in different solvents after 24 hours.

**Solvent**	**Solubility**
Water	Insoluble
Ethyl acetate	Insoluble
Hexane	Insoluble
p-Xylene	Insoluble
THF	Insoluble
DMSO	Insoluble
Ethanol	Insoluble
DCM	Insoluble

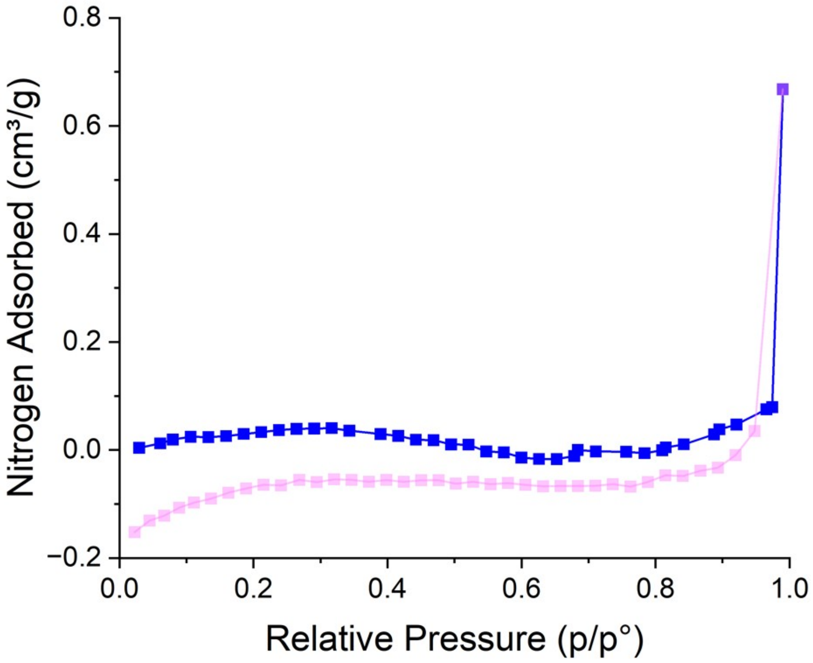

Figure S3. N_2 gas sorption isotherm of *poly*-PETMP showing a non-porous structure.

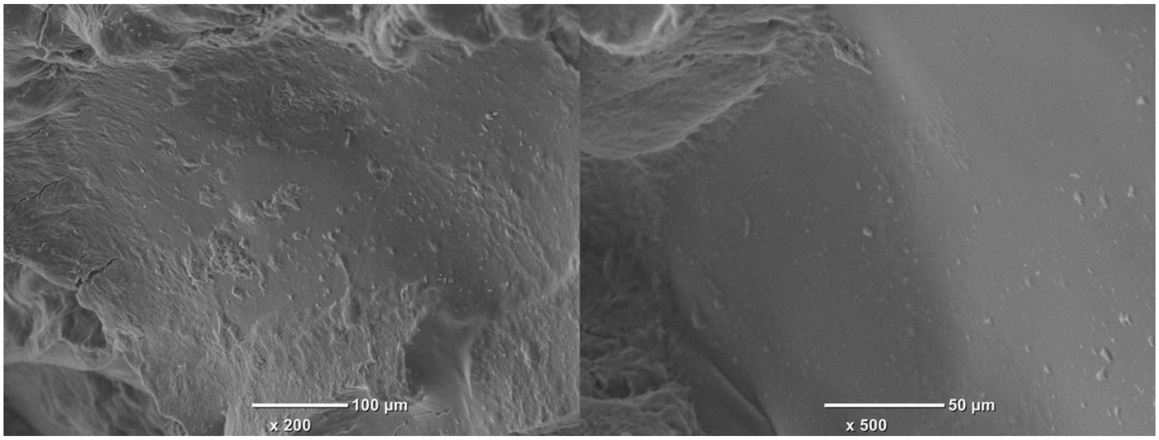

Figure S4. SEM imaging of poly-PETMP at x200 and x500 magnification.

Table S3. The measured swelling ratio of poly-PETMP in water, ethanol, and hexane, representing solvents of different polarity.

Solvent	Swelling percentage (%)
Water	29.1 ± 5.1
Ethanol	7.8 ± 6.9
Hexane	0 (-0.8) ± 1.3

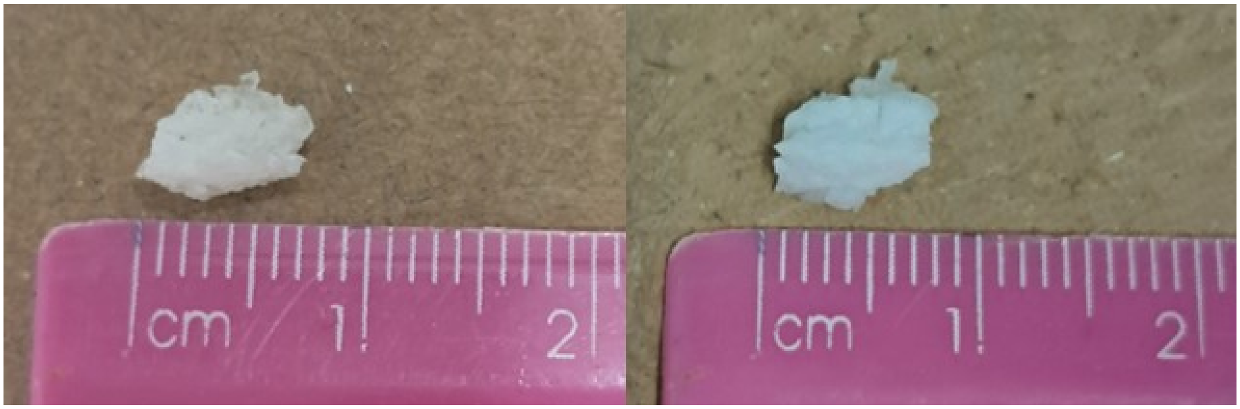

Figure S5. Pictures of poly-PETMP-Ca before (left) and after (right) exposure to an environment of 90% RH and 25 °C for 24 hours.

Table S4. A comparison of the AWH performance of *poly*-PETMP against other amorphous polymer networks reported, and Ca^{2+} loaded materials. We do not compare here to hydrogels or crystalline structures.

**Material**	**Network chemistry**	**BET surface area [m^2/g]**	**H_2O uptake at 0.3 P/P_0 [g/g]**	**Total uptake capacity [g/g]**	**Uptake T [°C]**	**Ref.**
			**Amorphous polymers**			
OHCP-60	Thermal oxidation	462	0.06	0.35	25	[1]
3D ep-POP	Oxirane (epoxide)	779	0.18	0.41	25	[2]
SHCP-10	$–SO_3H$	697	0.22	0.81	25	[3]
BM-1	S-S, $–SO_3H$	0	0.03	0.22	25	[4]
BM-3	S-S, $–SO_3H$	0	0.23	1.24	25	[4]
BM-5	S-S, $–SO_3H$	41	0.26	1.39 (1.60 after cycling)	25	[4]
S-SAS	C-S-C, $-SO_3Na$, $-S-S^-$	-	-	3.45	25	[5]
			**Ca^{2+} loaded materials**			
NJTech-4-Ca10	Zr-MOF, Ca^{2+}	538.7	0.61	-	25	[6]
$CaCl_2$@MOF-808	MOF-808, Ca^{2+}	1420	0.56	-	25	[7]
PNIPAM	GO, *poly*(N-isopropylacrylamide), Ca^{2+}	-	-	3.6	25	[8]
			**This work**			
Poly-PETMP	Sulfone, Sulfoxide, Ca^{2+}	0	0.29	1.34	25	This work

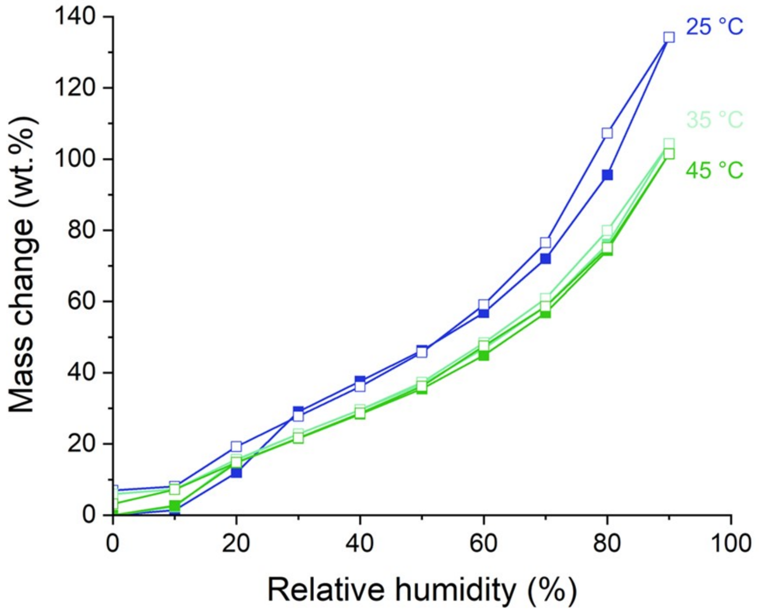

Figure S6. Overlay of the water sorption isotherms conducted at 25 °C, 35 °C, and 45 °C.

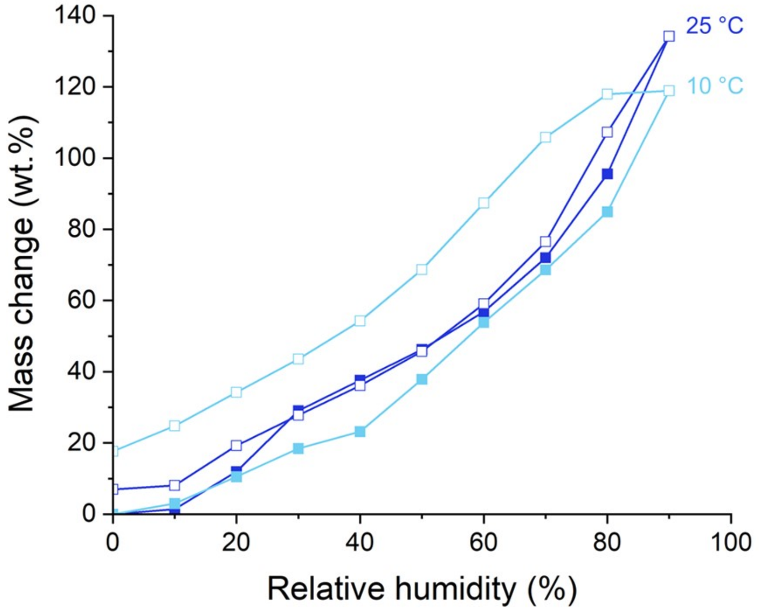

Figure S7. Overlay of the water sorption isotherms conducted at 10 °C and 25 °C.

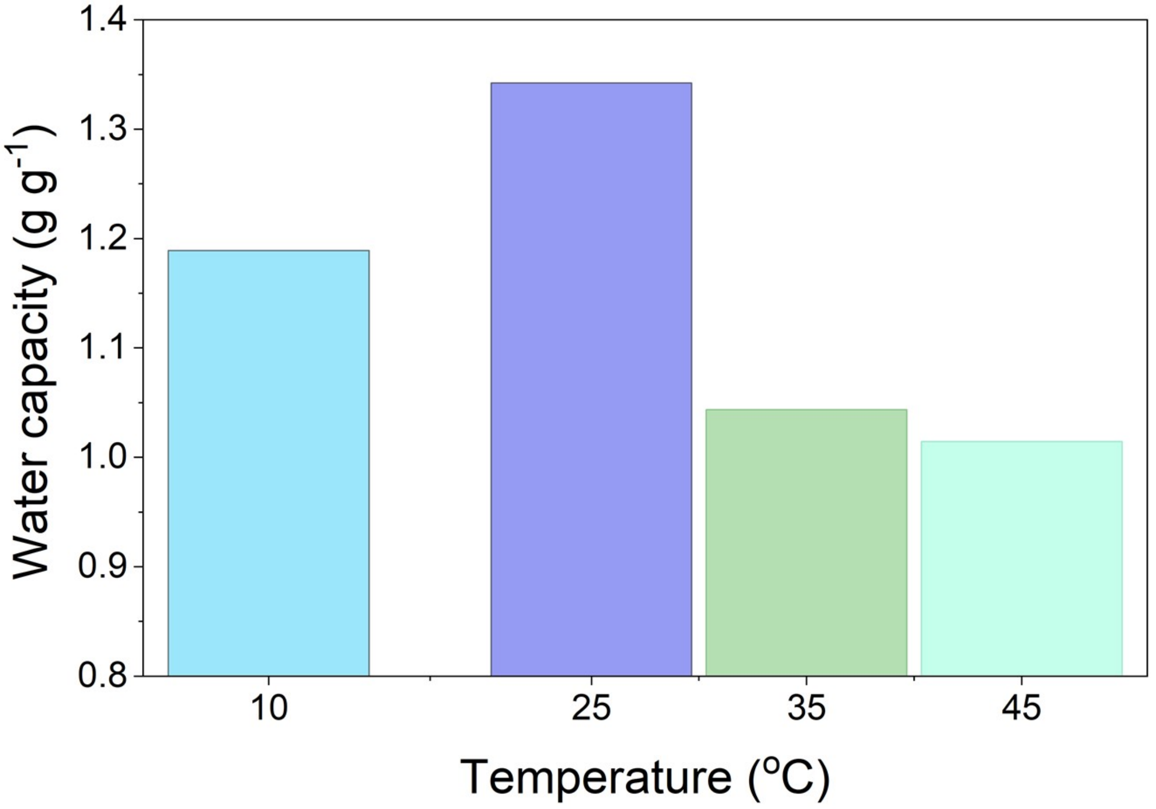

Figure S8. The maximum water sorption capacity measured by water sorption isotherm at 10 °C, 25 °C, 35 °C, and 45 °C.

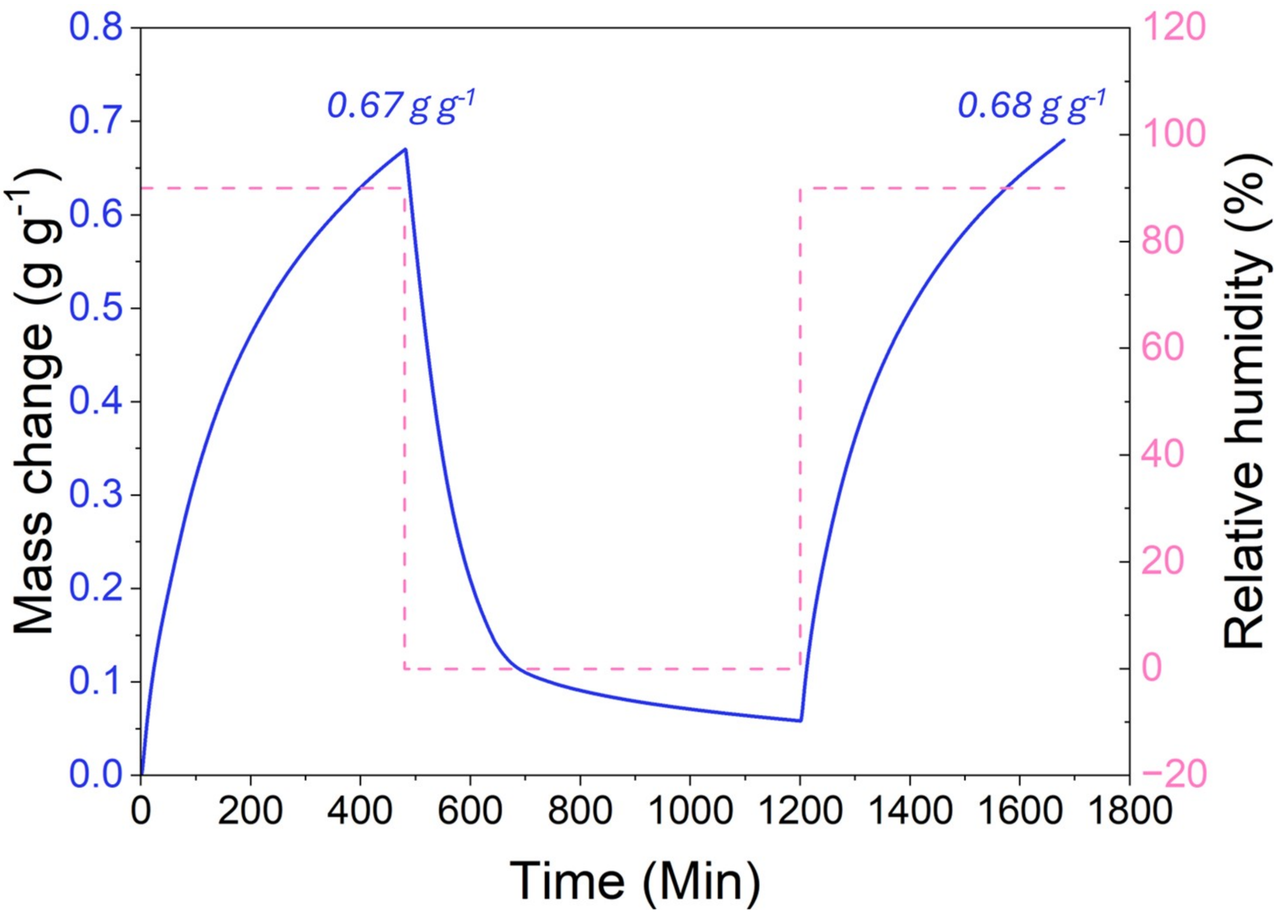

Figure S9. Absorption profile of poly-PETMP-Ca for 8 h at 90% RH after a 12 h drying procedure at 0% RH. A 12 h desorption profile followed by a second sorption profile at 90% RH for 8 h demonstrates no loss of capacity after sorption/desorption cycling at high RH.

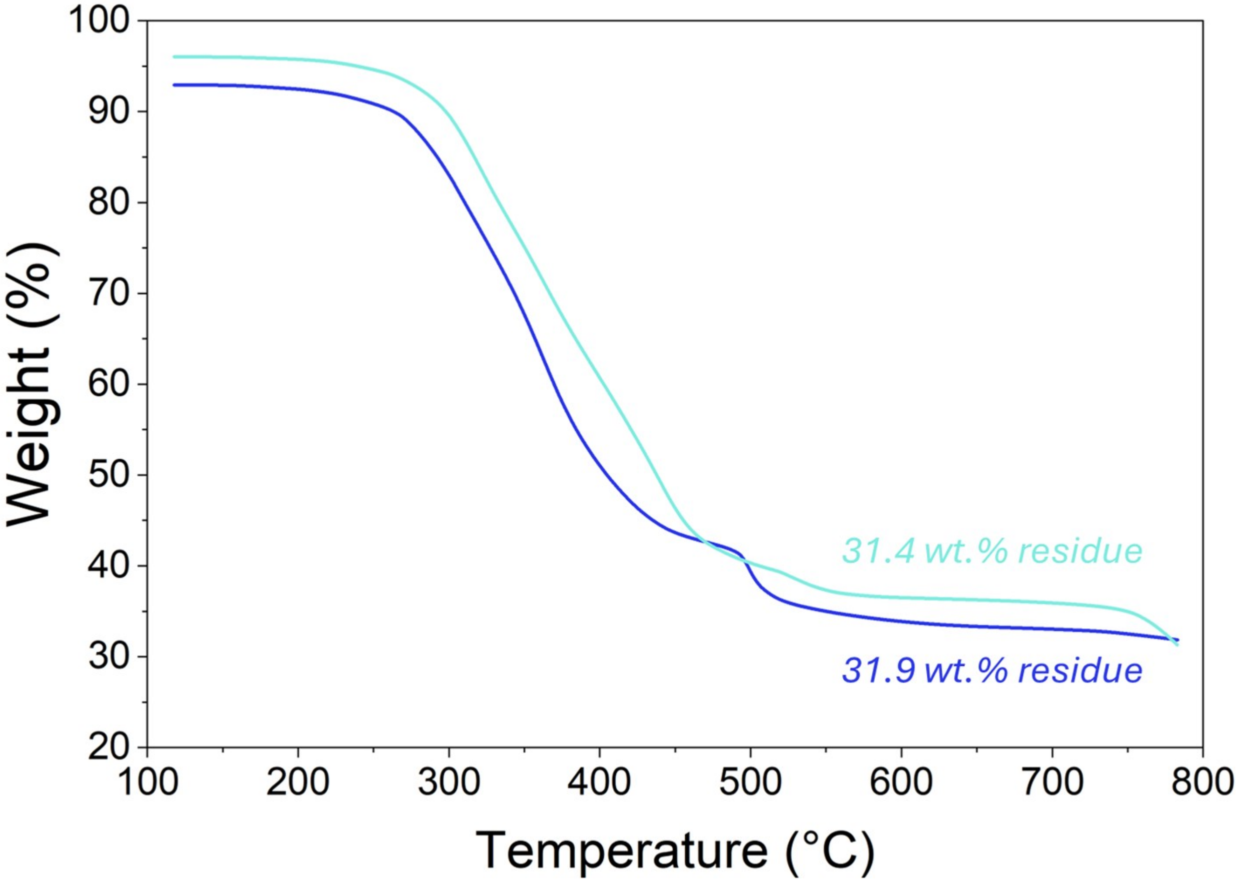

Figure S10. TGA thermograms of poly-PETMP-Ca before (blue) and after (teal) an absorption/desorption cycle at 75% RH and 25 °C.

References

[1] P. Schweng, L. Präg, R. T. Woodward, "Regulating the Hydrophilicity of Hyper-Cross-Linked Polymers via Thermal Oxidation for Atmospheric Water Harvesting", *ACS Appl. Mater. Interfaces,* **2024**, **16 (43)**, 58566–58572.

[2] Y. Byun, A. Coskun, "Epoxy-Functionalized Porous Organic Polymers via the Diels–Alder Cycloaddition Reaction for Atmospheric Water Capture", *Angew. Chem. Int. Ed.,* **2018**, **57 (12)**, 3173–3177.

[3] P. Schweng, F. Mayer, D. Galehdari, K. Weiland, R. T. Woodward, "A Robust and Low-Cost Sulfonated Hypercrosslinked Polymer for Atmospheric Water Harvesting", *Small,* **2023**, **19 (50)**, 2304562.

[4] J. J. Dale, P. Schweng, M. Gerbaud, R. T. Woodward, "Sulfonated, Disulfide-Bridged Polymer Networks for Atmospheric Water Harvesting", *Small*, 2026, e73271

[5] J. J. Dale, M. W. Smith, T. Hasell, "Water Soluble, Ionically Generated Thiopolymers", *Adv. Func. Mater.*, 2024, **32 (24)**, 2314567

[6] Y. Jiang, H. Tan, B. Wei, L. Du, J. Zhang, Z. Lu, "Hygroscopic salt in a mesoporous zirconium metal–organic framework for atmospheric water harvesting", *J. Mater. Chem. A*, 2025, **13**, 31469-31476.

[7] H. An, Y. Chen, Y. Wang, X. Liu, Y. Ren, Z. Kang, J. Li, L. Li, "High-performance solar-driven water harvesting from air with a cheap and scalable hygroscopic salt modified metal–organic framework", *Chem. Eng. J.,* 2023, **461**, 141955.

[8] X. Wang, D. Yang, M. Zhang, Q. Hu, K. Gao, J. Zhou, Z. Yu, "Super-Hygroscopic Calcium Chloride/Graphene Oxide/Poly(N-isopropylacrylamide) Gels for Spontaneous Harvesting of Atmospheric Water and Solar-Driven Water Release", *ACS Appl. Mater. Interfaces,* 2022, **14 (29)**, 33881–33891.
